# Prevalence of microvascular and macrovascular complications of diabetes in newly diagnosed type 2 diabetes in low-and-middle-income countries: A systematic review and meta-analysis

**DOI:** 10.1371/journal.pgph.0000599

**Published:** 2022-06-15

**Authors:** Faith Aikaeli, Tsi Njim, Stefanie Gissing, Faith Moyo, Uazman Alam, Sayoki G. Mfinanga, Joseph Okebe, Kaushik Ramaiya, Emily L. Webb, Shabbar Jaffar, Anupam Garrib

**Affiliations:** 1 Muhimbili Medical Research Centre, National Institute for Medical Research, Dar Es Salaam, Tanzania; 2 Department of International Public Health, Liverpool School of Tropical Medicine, Liverpool, United Kingdom; 3 School of Public Health Yorkshire & Humber, Leeds Teaching Hospitals NHS Trust, Leeds, United Kingdom; 4 Institute of Life Course and Medical Sciences and the Pain Research Institute, University of Liverpool and Liverpool University Hospital NHS Foundation Trust, Liverpool, United Kingdom; 5 Division of Endocrinology, Diabetes and Gastroenterology, University of Manchester, Manchester, United Kingdom; 6 Shree Hindu Mandal Hospital, Dar es Salaam, Tanzania; 7 Medical Research Council International Statistics and Epidemiology Group, London School of Hygiene and Tropical Medicine, London, United Kingdom; 8 Department of Clinical Sciences, Liverpool School of Tropical Medicine, Liverpool, United Kingdom; University of Embu, KENYA

## Abstract

There is an excessive burden of diabetes complications in low-resource settings. We conducted a systematic review to determine the nature and frequency of diabetes complications in newly diagnosed with type 2 diabetes. A systematic search was performed using Medline, CINAHL and Global Health online databases from inception to July 2020. Articles reporting prevalence of microvascular or macrovascular complications within six months of type 2 diabetes diagnosis and published in English or French from low- and middle-income countries (LMICs) were eligible for analysis. Data were extracted using a standardized data extraction tool. Descriptive statistics were used to describe the prevalence of micro and macrovascular complications in newly diagnosed type 2 diabetes. Assessment of heterogeneity was conducted using the inconsistency index (I^2^) and Cochran-Q chi^2^ statistical tests. Publication bias was assessed by the Funnel plot and Egger test. A total of 3 292 records underwent title or abstract screening and 95 articles underwent full text review. Thirty-three studies describing 13 283 participants (aged 20 years and older) met the inclusion criteria. The eligible studies were from Asia (n = 24), Africa (n = 4), Oceania (n = 2), South America (n = 2) and the Caribbean (n = 1). For microvascular complications, the median prevalence (interquartile range) of retinopathy, nephropathy and neuropathy were 12% (6%-15%), 15% (7%-35%) and 16% (10%25%) respectively. For macrovascular complications, the median prevalence (interquartile range) was 10% (7%-17%) for ischaemic heart disease, 6% (1%-20%) for peripheral arterial disease and 2% (1%-4%) for stroke. There was evidence of substantial heterogeneity between studies for all outcomes (I^2^ > 90%. We found a high prevalence of complications in newly diagnosed type 2 diabetes in LMICs. Findings suggest that many people live with diabetes and are only diagnosed when they present with complications in LMICs. Research is needed to guide timely and effective identification of people living with diabetes in these settings.

## Introduction

Globally an estimated 463 million adults aged 20–79 years are currently living with diabetes. This number is expected to increase by 51% to 700 million by 2045 [1]. Type 2 diabetes mellitus is the most common form of diabetes, representing about 90% of all diabetes cases worldwide [2]. It is characterised by a long asymptomatic period of five to seven years from onset to diagnosis, such that many patients present with complications at the time of diagnosis [3]. Within the first ten years from diagnosis, an estimated 27% of people with type 2 diabetes die [4].

Microvascular and macrovascular complications are the major cause of morbidity and mortality in people with diabetes [2]. Macrovascular complications include myocardial infarction, stroke, peripheral vascular disease and diabetic foot. There is an increase in five-year mortality in patients diagnosed with macrovascular complications [5]. Microvascular complications of type 2 diabetes include retinopathy, nephropathy and neuropathy [6] of which there is an excess burden in persons newly diagnosed with type 2 diabetes [7]. Importantly, newly diagnosed diabetes is associated with substantial premature death not only from vascular disease but also other non-vascular causes of mortality [8].

Studies characterising recent and long-term trends in diabetes-related complications globally are based on data predominantly coming from high-income countries [9, 10]. Low- and middle- income countries (LMICs) are the focus of this study as they are home to 79% of adults with diabetes, and there is a paucity of evidence synthesis detailing the burden of diabetes complications in these settings [1].

We sought to systematically review the literature on both microvascular and macrovascular complications at presentation among patients diagnosed recently with type 2 diabetes in LMICs.

## Materials and methods

This review followed a protocol which was registered in the PROSPERO database (CRD42019126762; available from https://www.crd.york.ac.uk/prospero/display_record.php?ID=CRD42019126762. The findings have been reported according to the Preferred Reporting Items for Systematic Reviews and Meta-Analysis (PRISMA) guidelines (S1 Checklist).

### Search strategy and selection criteria

We searched the following online databases: Medline, CINAHL and Global Health using predefined search strategies for relevant abstracts. The main search terms included: “Diabetes mellitus” “newly diagnosed” “microvascular complications” and “macrovascular complications” (S1 Text).

All articles reporting complications at diagnosis (regardless of diagnostic method, definition or classification) published in English or French and from LMICs, up to July 2020 were included. Countries with a gross national income below US $12 376 defined LMICs according to the World Bank Country and Lending Groups classification [11].

Case series, studies with a sample size of less than 30 participants, letters to the editor, reviews, editorials, commentaries, conference abstracts of unpublished studies and studies including participants with gestational, or type 1 diabetes were excluded. Cross sectional, cohort and case control studies, case series with > 30 patients and randomised control studies were included. For multiple studies presenting results of the same population, the study with the most complete data was included. For studies with several publications of findings over time, the most recent was included.

### Data extraction

Articles returned by the search were saved to EndNote software which was used to remove duplicates. Titles and abstracts of the articles obtained were subsequently assessed for eligibility using the inclusion and exclusion criteria by two reviewers working independently.

Full text articles were then retrieved and assessed by two reviewers independently (TN, SG, FA and EW), and their references were also screened. Disagreements were resolved by discussion with a third author (OJ). Full texts of articles that could not be retrieved or articles where important information was missing were requested from corresponding authors through emails. Reminders were sent weekly, and the articles were excluded if no response was obtained after a month.

### Data management

An *a priori* data extraction tool was created on Microsoft Excel 2010 and pre-tested. Following full text screening, data were extracted into the tool by two independent reviewers (TN, SG, FA and EW). A third author (AG) checked that the data was correct and resolved discrepancies by discussion. The following information was extracted: surname of first author; date of publication; country; region; study design; definition used for diagnosis of type 2 diabetes; age range of participants; health facility type; various microvascular and macrovascular complications; their diagnostic criteria and their respective proportions in the participants.

### Assessment of risk of bias

Included studies were assessed for methodological quality and risk of bias using the Quality Assessment Tool for Observational, Cohort and Cross-Sectional Studies of the National Health Institute (S1 Table) by two independent reviewers (FA and EW). Studies were classified to have either “good”; “fair” or “poor” quality.

### Data synthesis and analysis

Preliminary checks for heterogeneity were performed to assess the possibility of combining evidence from primary studies in a meta-analytic approach. The Cochran-Q chi^2^ statistical test for heterogeneity was used to assess interstudy variability while the inconsistency statistic (I^2^) quantified the proportion of between study heterogeneity with values of 0%-25%, 50%-75%, >75% representing low, medium, and substantial heterogeneity, respectively [12]. Due to the substantial heterogeneity observed between studies (I^2^ >90%), combining available evidence in a meta-analytic approach was not feasible. Therefore, descriptive statistics (median, range and interquartile range) were used to describe the prevalence of study outcomes from primary studies. Prevalence data for the individual studies were summarised in Forest plots.

Where possible, subgroup analyses were conducted to explore sources of heterogeneity, i.e. whether prevalence of a complication varied with the following prespecified study characteristics: region in which the study was conducted, gender, age, criteria for diagnosis, and type of health facility. For this subgroup analysis, random effects meta-analysis models were fitted to estimate the pooled prevalence and associated 95% confidence interval for each chronic complication within each subgroup. The selection of these covariates was guided by their clinical or evidence-based relevance. A small number of characteristics was chosen to reduce the likelihood of false positive results. Evidence for publication bias was assessed graphically by creating funnel plots from the inverse variance of the proportion of newly diagnosed type 2 diabetes with various complications. Statistical tests for the funnel plot asymmetry were done using the Egger test while the non-parametric trim and fill tests were conducted to account for potentially missing studies.

## Results

### Search results

**Fig 1:**
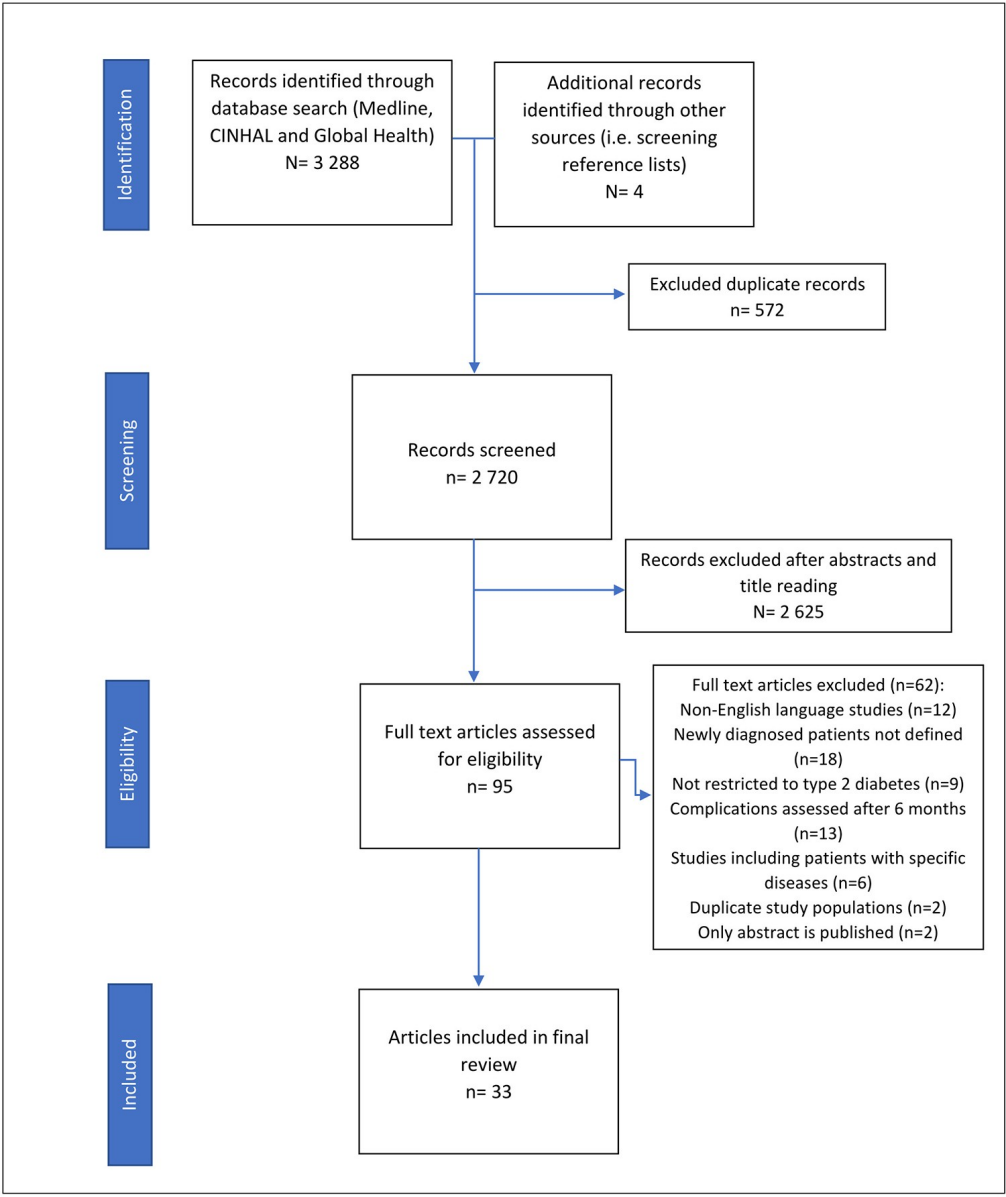
Prisma flow chart of the systematic review and article selection.

Database searches yielded 3 288 articles and four additional articles were identified from the reference list of which, 2 720 remained after duplicate removal. Titles and abstracts of candidate articles were screened to exclude 2 625 articles leaving 95 records for full text screening. Sixty-two articles were excluded for the following reasons: 18 studies did not identify newly diagnosed patients, 13 studies assessed complications after six months, 12 studies were in other languages, nine studies included patients with type 1 diabetes and in six studies the study population were patients with specific diseases. Two studies included data from the same population, one study only had an abstract published and one author did not respond to queries for full texts. A total of 33 studies met the inclusion criteria and were included in the final review (Fig 1).

### Study characteristics

The characteristics of the 33 studies included in the final review are described in Table 1 [2–4, 9, 10, 13–40]. The studies reported on a total of 13 283 (aged ≥20 years) participants from fourteen countries belonging to LMIC group as per the World Bank classification criteria [11]. Among these studies, 31 were cross sectional [2–4, 9, 10, 13–37, 40], one a cohort [38] and one a case-control study [39]. More than two thirds of the studies, 24 (73%), were conducted in Asia [2, 3, 9, 14–16, 18, 19, 21, 23, 25–29, 31–35, 37–39] while only four were from Africa [10, 13, 30, 36], two from Oceania [17, 20], two from South America [22, 24] and one from the Caribbean [4]. A detailed assessment of the studies included in this review was carried out and the risk scores for bias are presented in S1 Table.

**Table 1:** Characteristics of studies describing proportions of newly diagnosed type 2 diabetic patients with different microvascular and macrovascular complications.

Complication	Number of studies	Location of studies	Number of participants	Median prevalence (IQR)
**Retinopathy**	22	Asia– 20	10 427	12% (6%-15%)
		Africa– 1		
		Oceania—1		
**Nephropathy**	22	Asia– 17	10 409	15% (7%-35%)
		Africa– 2		
		Oceania– 2		
		South America—1		
**Microalbuminuria**	11	Asia– 7	2 276	24% (12%-44%)
		Africa– 1		
		Oceania– 2		
		South America—1		
**Macroalbuminuria**	8	Asia– 5	7 180	6% (4%-24%)
		Oceania– 2		
		South America—1		
**Neuropathy**	17	Asia– 14	9 701	16% (10%-25%)
		Africa– 2		
		South America—1		
**Myocardial Infarction**	1	Sri Lanka	597	7%* (6% - 10%)
**Ischaemic Heart Disease**	10	Asia– 10	8 418	10% (7%-17%)
**Peripheral Arterial Disease**	6	Asia– 4	2 041	6% (1%-20%)
		Africa– 1		
		South America– 1		
**Stroke**	4	Asia– 4	2 332	2% (1% - 4%)
**Diabetic Foot**	2	Asia -2	105	1%* (0%-1%)

**Abbreviation(s):** IQR interquartile range.

*Proportion (95% confidence interval) of participants with myocardial infarction or diabetic foot, reported by one study.

**Fig 2:**
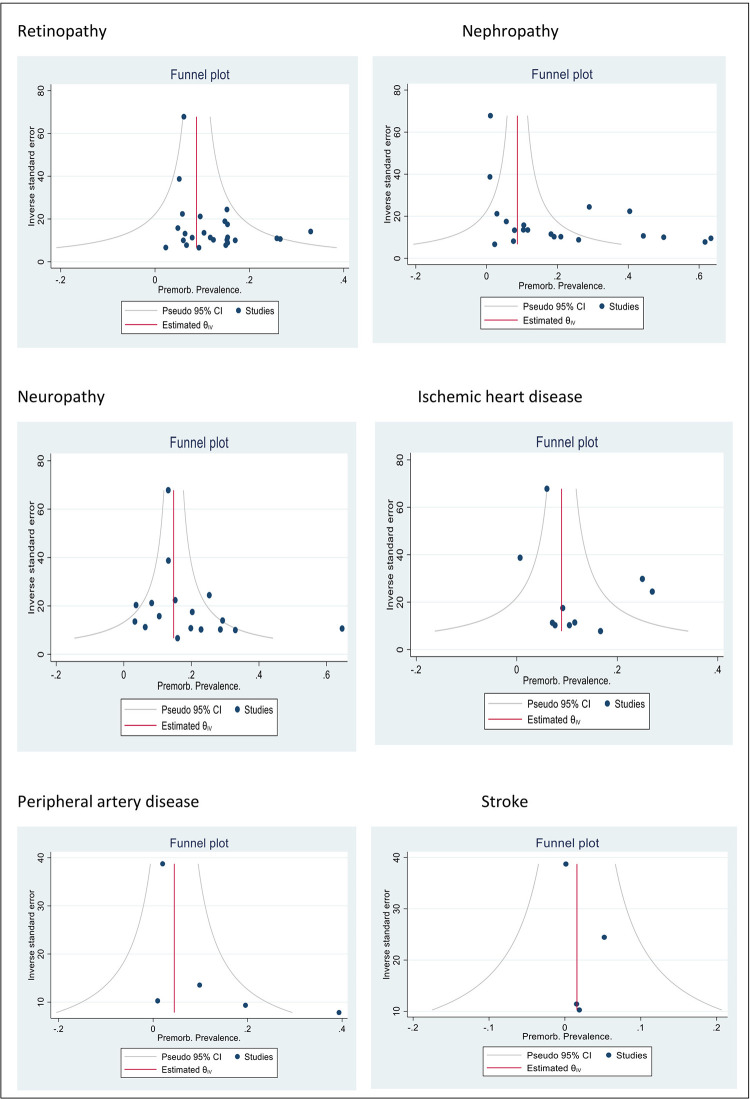
Evidence for publication bias by type of complication from diabetes.

Ten studies were rated to be of “good” quality [13–22], seventeen were given a “fair” quality rating [2–4, 9, 10, 23–34] and six studies were given a rating of “poor” quality [35–40]. Publication bias was assessed graphically by a funnel plot and the Egger test (Fig 2).

### Diabetes complications

#### a) Retinopathy

**Fig 3:**
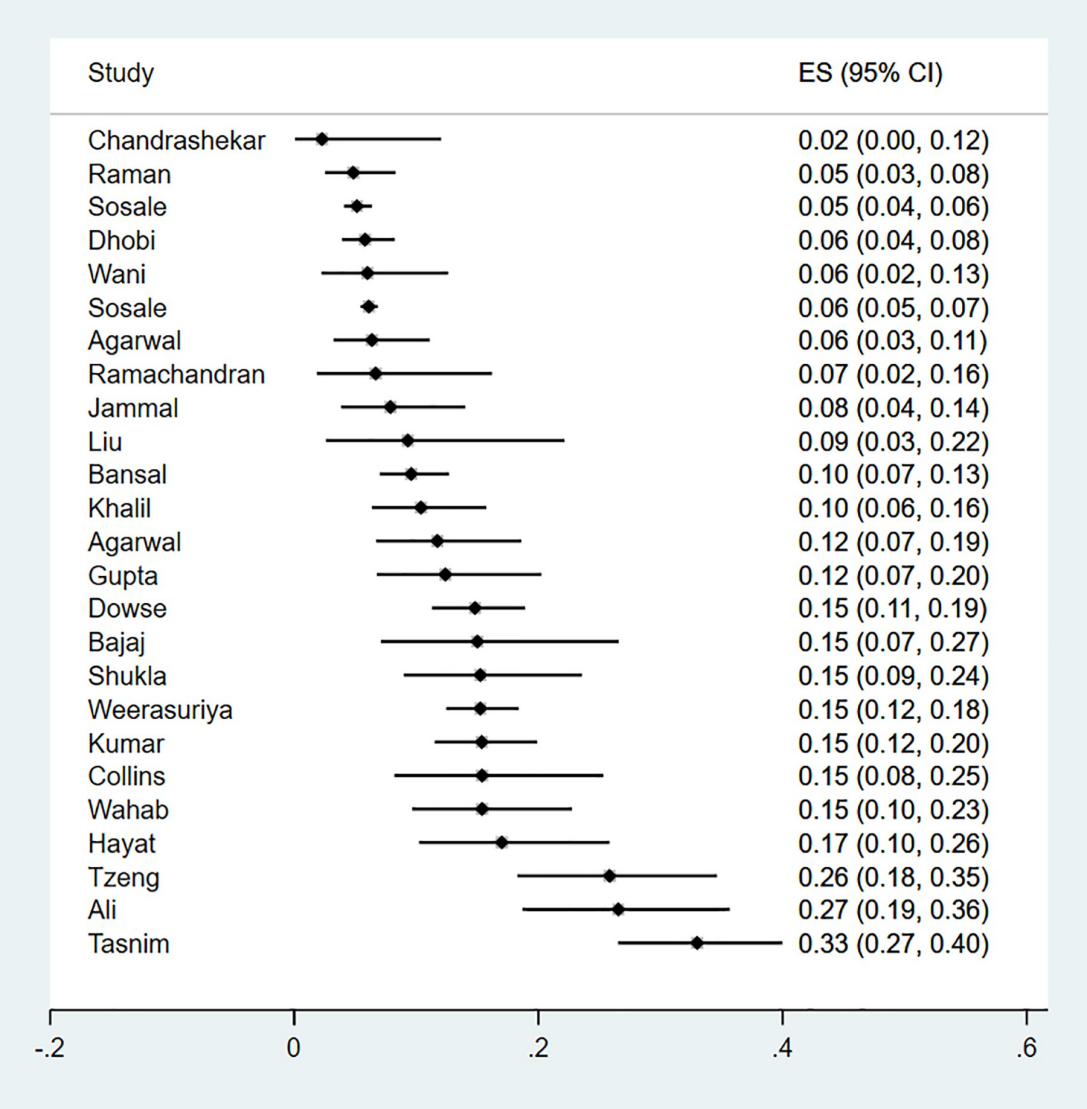
Forest plot illustrating the prevalence of retinopathy among newly diagnosed type 2 diabetes mellitus patients in low- and middle-income countries.

This microvascular complication was reported in 22 studies which enrolled a total of 10 427 newly diagnosed type 2 diabetes. Characteristics of these studies and methods used for diagnosis are described in S2 Table. The majority of studies were from Asia (n = 20), one study was from Africa and another was from Oceania. The prevalence of diabetic retinopathy ranged from 2%-33% (Fig 3), with studies documenting the lowest and highest prevalence both from Asia.

**Fig 4:**
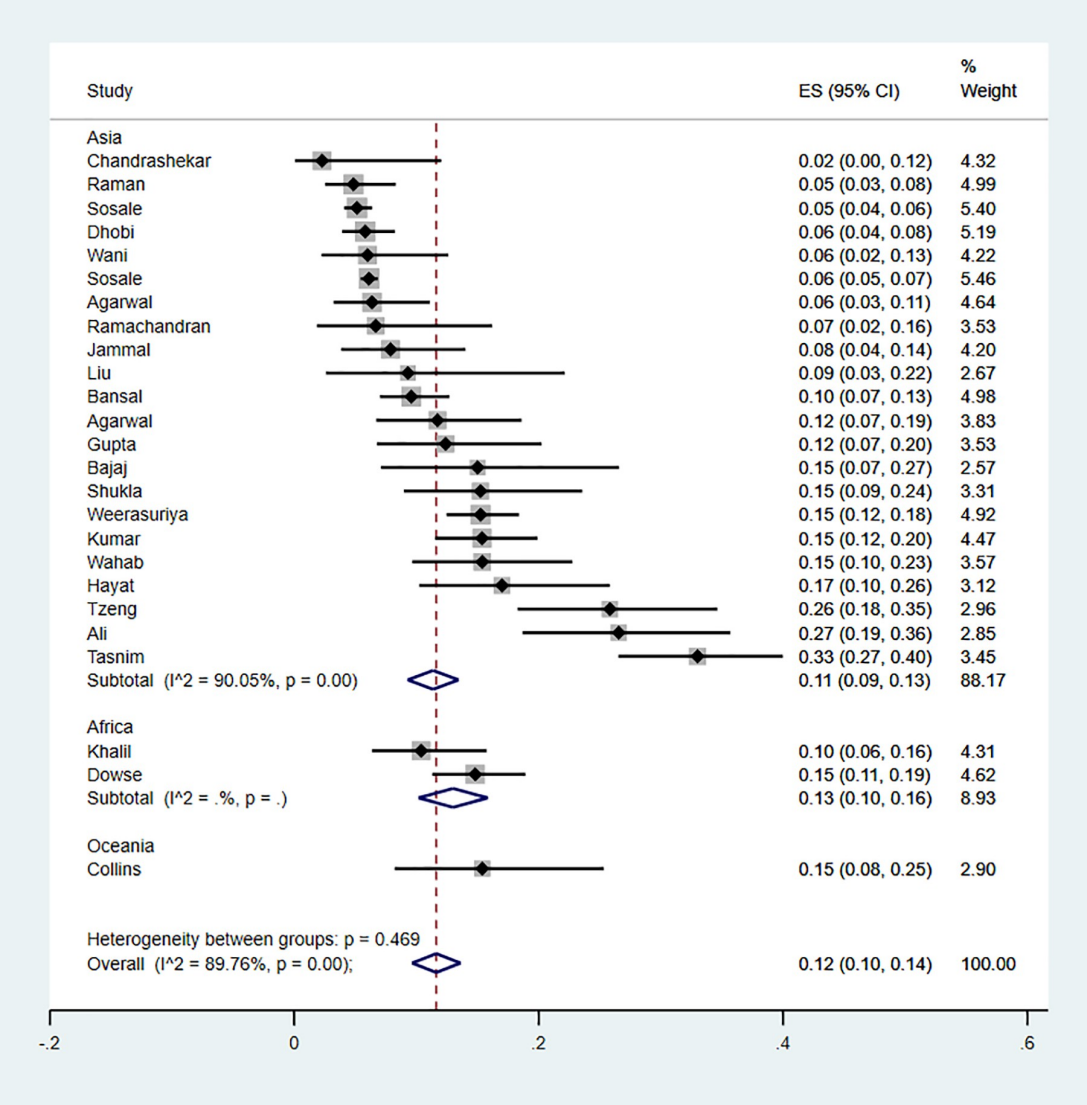
Forest plot illustrating the prevalence of retinopathy among newly diagnosed type 2 diabetes mellitus patients in low- and middle-income countries, by region.

When stratified by study region, the pooled prevalence of retinopathy in Asia was 11% (9%-13%), 13% (10%-16%) in Africa and 15% (8%-25%) in Oceania (P value = 0.469) suggesting that regional differences did not account for the heterogeneity observed (Fig 4). Further analyses were impossible to conduct due to sparsity of data. Publication bias assessed by the Egger test for symmetry showed significant results (P = 0.002) suggesting possible publication bias (Fig 2).

#### b) Nephropathy

**Fig 5:**
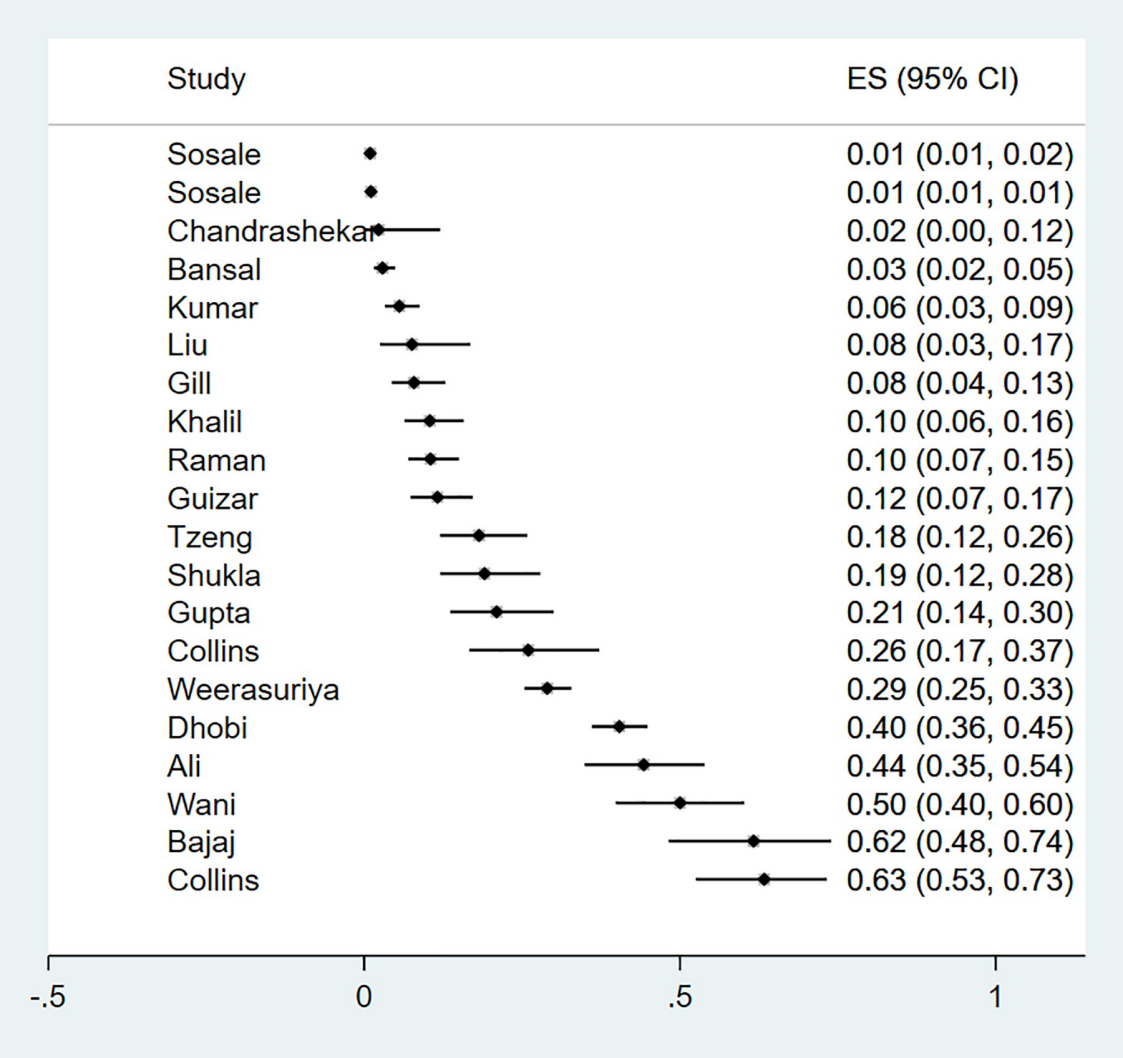
Forest plot illustrating the prevalence of nephropathy among newly diagnosed type 2 diabetes mellitus patients in low- and middle-income countries.

Nephropathy was reported in 22 studies (n = 10 409). The majority of studies were from Asia (n = 17), two studies were from Africa, two from Oceania and one study was from South America (Table 1). Characteristics of the studies and methods used for the diagnosis of nephropathy are described in S3 Table. The prevalence of nephropathy ranged from 1% to 63% among the eligible primary studies (Fig 5).

**Fig 6:**
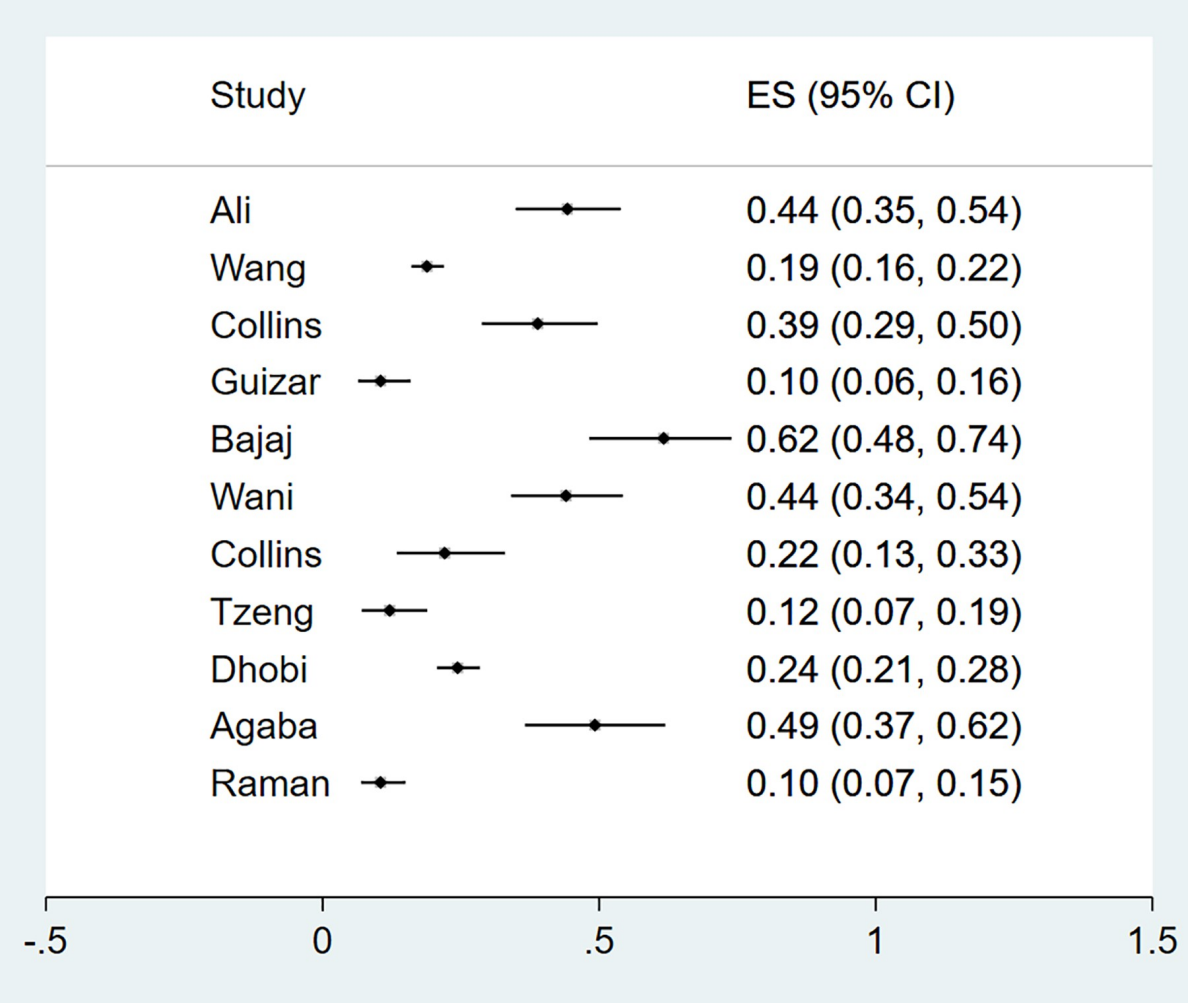
Forest plot of the prevalence of microalbuminuria among newly diagnosed type 2 diabetes mellitus patients in low- and middle-income countries.

**Fig 7:**
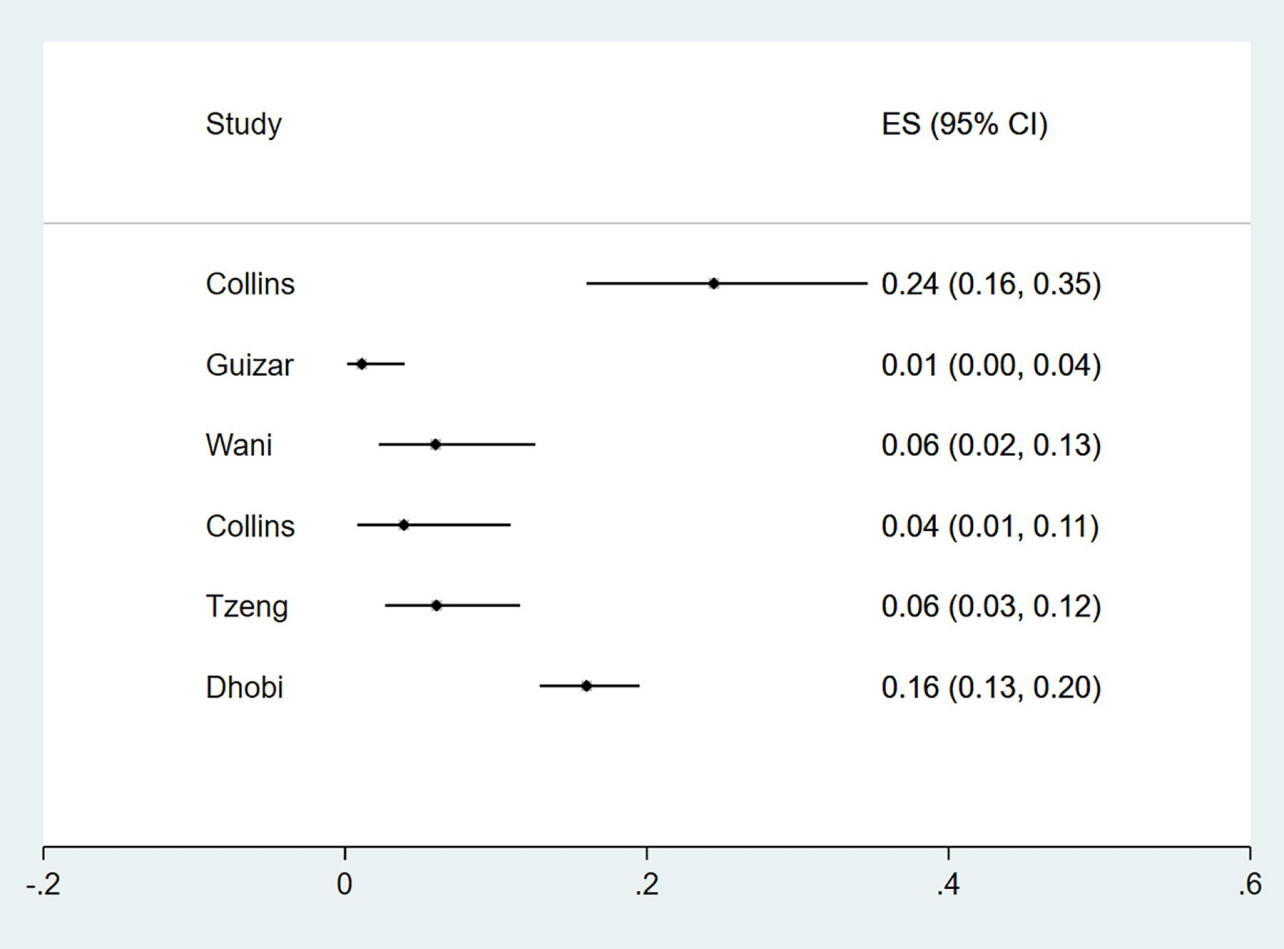
Forest plot of the prevalence of macroalbuminuria among newly diagnosed type 2 diabetes mellitus patients in low- and middle-income countries.

Eleven studies reported microalbuminuria with prevalence ranging from 10% to over 50% among eligible studies (Fig 6). Macroalbuminuria was reported in eight studies and the prevalence of this condition among primary studies ranged from 1%-24% (Fig 7). Further subgroup analyses were not feasible due to the different criteria used to define nephropathy and the paucity of studies in different categories. The funnel plot was asymmetrical, and this was supported by a statistically significant p value of the Egger test (P = 0.049) suggesting possible publication bias (Fig 2).

#### c) Peripheral neuropathy

The prevalence of diabetic neuropathy was reported in 17 studies (n = 9 701). Fourteen of the studies were from Asia, two were from Africa and one from the Caribbean. The characteristics of the studies and methods used to diagnose neuropathy are summarised in S4 Table.

**Fig 8:**
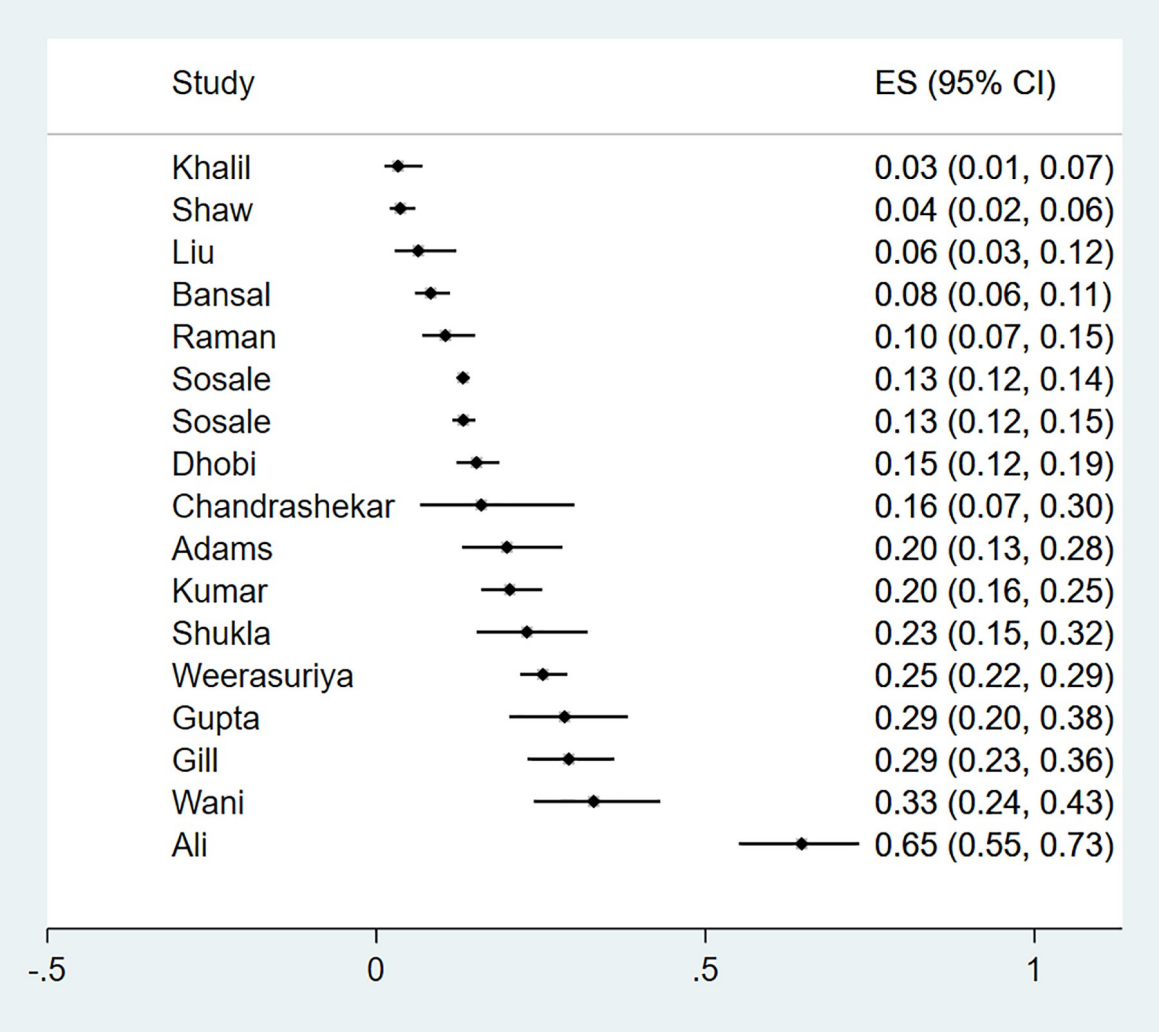
Forest plot illustrating the prevalence of neuropathy among newly diagnosed type 2 diabetes mellitus patients in low- and middle-income countries.

**Fig 9:**
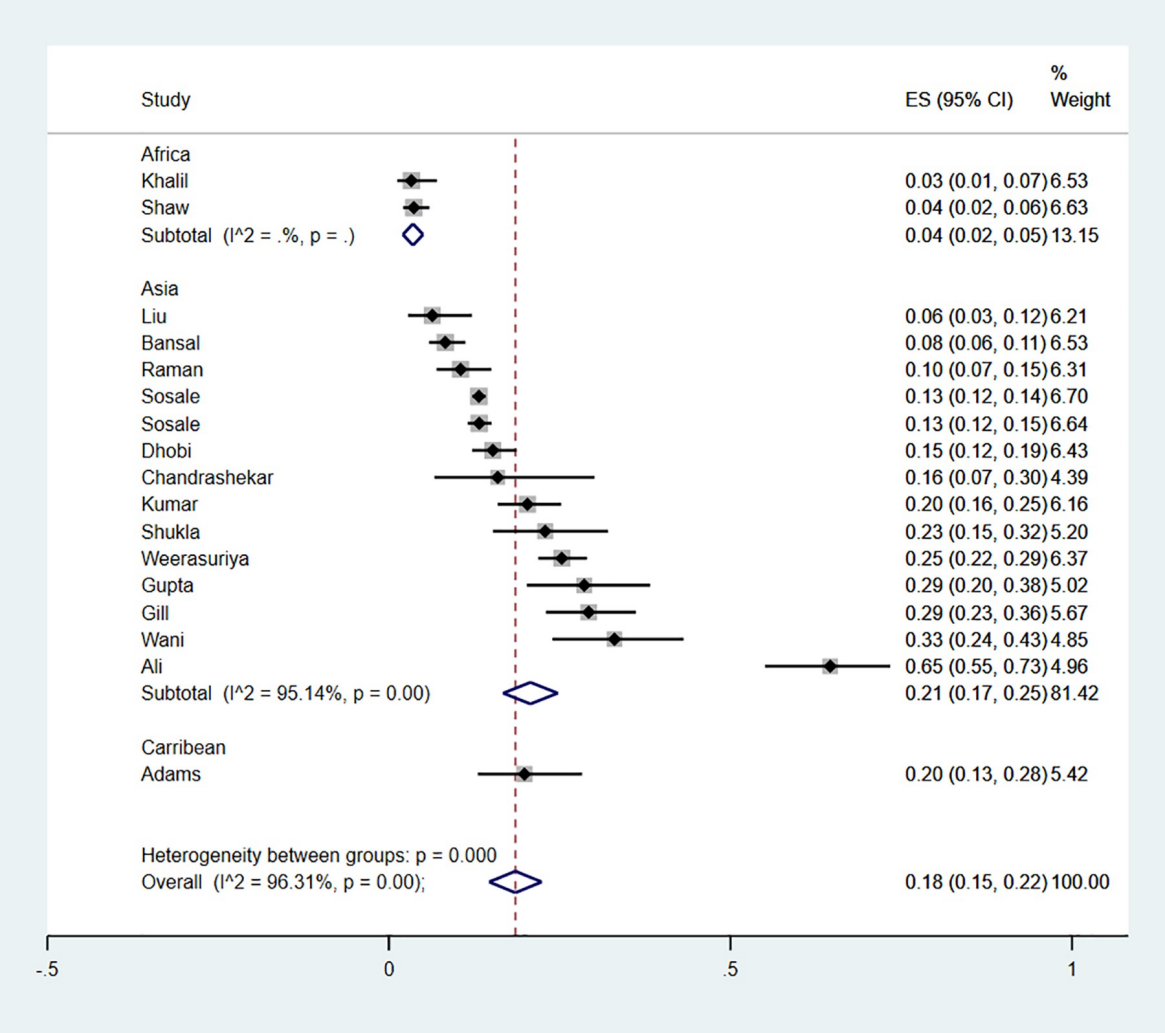
Forest plot illustrating the prevalence of neuropathy among newly diagnosed type 2 diabetes mellitus participants in low- and middle-income countries, by region.

The prevalence of neuropathy ranged from 3% to 65% (Fig 8). Sub-group analysis by region gave a pooled prevalence of 4% (4%-5%) for the two studies conducted in Africa, 20% (14%-28%) for a study from the Caribbean and 21% (17%-25%) for 14 studies conducted in Asia. The p value for the difference between subgroups was statistically significant (p<0.01), suggesting that the region in which the study was conducted accounted for some of the heterogeneity observed (Fig 9). A funnel plot did not demonstrate evidence of publication bias (Egger test, p = 0.11) (Fig 2).

#### d) Myocardial Infarction (MI)

The proportion of participants with MI at diagnosis of type 2 diabetes was reported by Weerasuriya et al. [27]. This study was conducted in a Sri Lankan specialised diabetic clinic and the diagnostic criteria were symptoms suggestive of MI. A total of 597 participants were screened. Forty-four participants had symptoms suggestive of MI, representing a MI prevalence of 7% (5%-10%).

#### e) Ischaemic Heart Disease (IHD)

**Fig 10:**
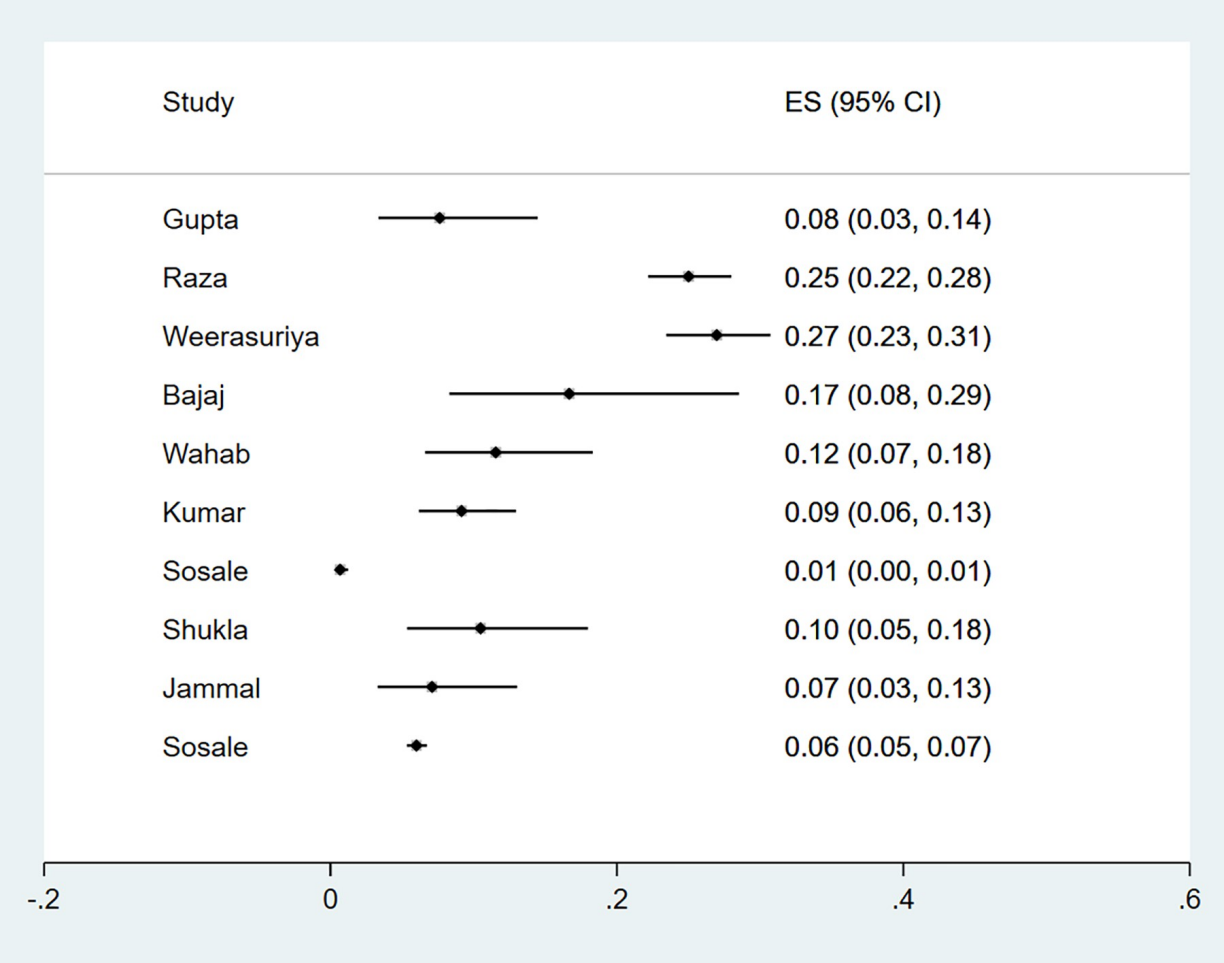
Forest plot of the prevalence of IHD among newly diagnosed type 2 diabetes mellitus patients in low- and middle-income countries.

The proportion of participants presenting with IHD was reported in ten studies (n = 8 418), S5 Table describes characteristics of these studies and criteria used for the diagnosis of IHD. The prevalence of IHD in newly diagnosed type 2 diabetes ranged from 1%-27% (Fig 10) with substantial heterogeneity. The funnel plot did not demonstrate evidence of publication bias (Egger test, P = 0.98) (Fig 2).

#### f) Peripheral Arterial Disease (PAD)

An estimate for newly diagnosed type 2 diabetic patients presenting with PAD at diagnosis were reported in six studies. Four of the studies were from Asia, one was from Africa and one from South America. A total of 2 041 participants were studied and characteristics and criteria for diagnosis are summarised in S6 Table.

**Fig 11:**
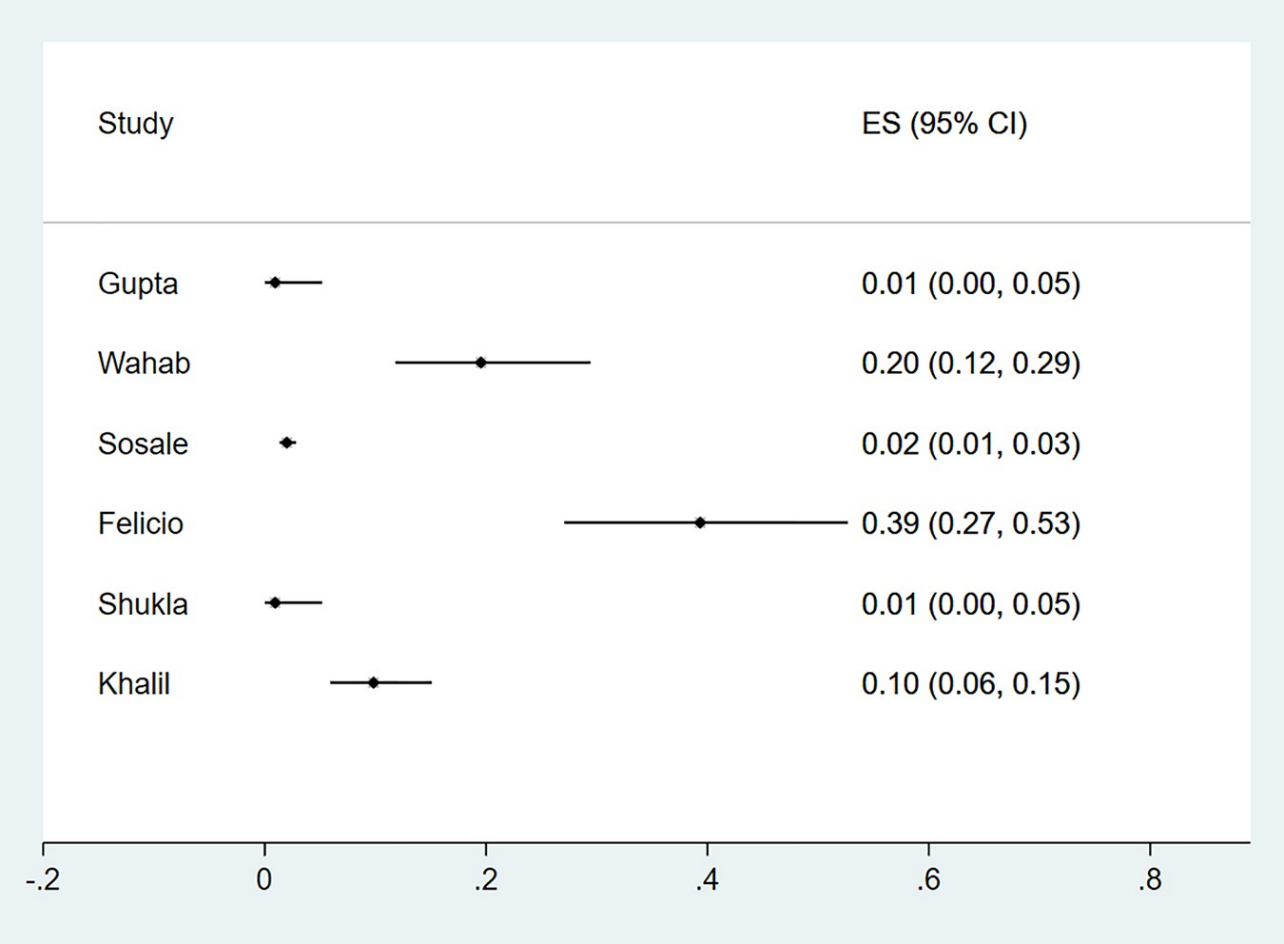
Forest plot illustrating the prevalence of peripheral arterial disease among newly diagnosed type 2 diabetes mellitus patients in low- and middle-income countries.

The prevalence of PAD in newly diagnosed type 2 diabetic participants ranged from 1%-40% (Fig 11) with substantial heterogeneity. Funnel plot symmetry was used to assess the risk of publication bias for which there was no evidence (Egger P value = 0.09) (Fig 2).

#### g) Stroke

**Fig 12:**
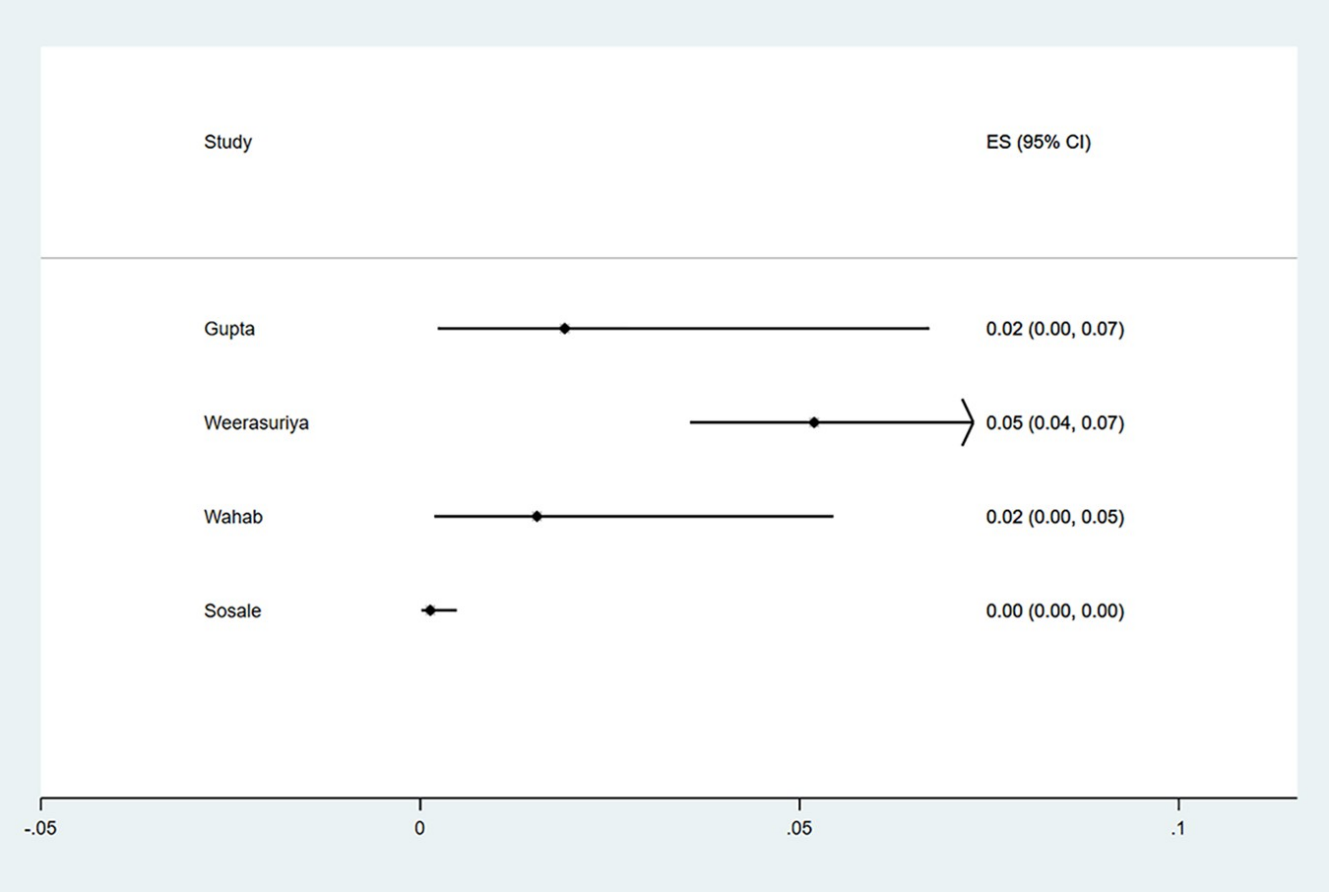
Forest plot of the prevalence of stroke among newly diagnosed type 2 diabetes mellitus patients in low- and middle-income countries.

Four studies reported the proportion of participants presenting with stroke at the time of diagnosis of type 2 diabetes. All studies were conducted in Asia (n = 2,332 patients). The study characteristics and criteria for diagnosis of stroke are summarized in S7 Table. The reported prevalence ranged from 0% to 5% (Fig 12), with no evidence of publication bias (Egger P value = 0.72) (Fig 2).

#### h) Diabetic foot

The proportion of newly diagnosed type 2 diabetes patients presenting with diabetic foot disease was reported by two studies carried out in Asia. Gupta et al. [3] studied 105 patients in a tertiary hospital in India and one patient was reported to have diabetic foot at first diagnosis. Chandrashekar et al. [38] studied 44 patients and found no patients presenting with diabetic foot at time of diagnosis. Characteristics of these studies are summarised in S8 Table.

## Discussion

### Summary of the findings

This systematic review reports on the burden of diabetes complications at the time of diagnosis of type 2 diabetes in LMICs.

We found that the prevalence of microvascular complications in newly diagnosed type 2 diabetes was estimated at 12% (IQR: 6%-15%) for retinopathy. Diabetic retinopathy is one of the leading causes of blindness among working age adults around the world [41] The median prevalence of retinopathy in our study was higher than that reported from studies done in high income countries like Denmark (6.8%) [42] in the Netherlands (0.7%) [43] and South Korea (2.8%) [44] but was lower than the 18% found in two studies in the UK [45, 46]. These differences could reflect retinopathy screening and diagnostic methods and definitions, early identification of people with type 2 diabetes and other health systems and individual level factors [46].

The median proportion of patients presenting with nephropathy was estimated at 15% (IQR: 7%-35%) in this study and is higher than that reported in high income countries. It is twice the reported estimate in the United Kingdom (7.2%) [47] and Netherlands (12.4%) [43] and six times that reported in Denmark (3%) [41]. Similarly, in this review we found a prevalence of diabetic neuropathy of 16%. The prevalence reported in high-income settings is much lower for patients presenting at diagnosis ranging from 2.3 to 8.2% in the United Kingdom [48, 49], 9.8% in Northern Ireland [50], 8.7% in Finland [51], 6.3 in Germany [52], 6% in Australia [53], 4% in Denmark [54] and 1.7 to 3.9% in the Netherlands [43].

The median prevalence of macrovascular complications in newly diagnosed type 2 diabetes was lower compared to microvascular complications, with estimated prevalence ranging between 6%-10% for peripheral arterial disease, myocardial infarction, and ischemic heart disease. The median prevalence of stroke and the diabetic foot in the eligible studies was low at 2% (IQR: 1%-4%) and 1% (0%-1%) respectively. Macrovascular complications such as cardiovascular diseases are the leading cause of disability and death among individuals with diabetes, accounting for approximately half the deaths globally [55]. However, recent data from high-income countries demonstrates that rates of myocardial infarction, stroke and amputation are decreasing among people with diabetes, in parallel with declining mortality [56], possibly as a result of earlier diagnosis and more aggressive management of diabetes and other co-morbidities that may contribute to the development of these complications. The prevalence of macrovascular complications in this review were higher than those reported in high-income settings. The prevalence of IHD in the US is 7.5% and of stroke is 1.7%. In Europe the Discover study reported a prevalence of 3.1% for peripheral arterial disease [57]. There are very few studies that explored macrovascular complications in LMICs, hence these complications are less well understood. The current understanding of the international burden of and variation in diabetes-related complications is poor [56], a knowledge gap which this study attempted to narrow.

Our findings demonstrate a considerable burden of diabetes complications at time of diagnosis in LMICs. The findings may be due to a lack of or inaccessibility of services, resulting in delays in seeking care among people living with diabetes in LMICs compared to individuals from high-income settings. In high-income countries, a steady decline in all-cause mortality rates and in the incidence of complications in persons with type 2 diabetes has been seen [58]. The lower prevalence observed in high-income countries is likely attributable to an enabling policy environment which facilitates early detection of type 2 diabetes, resulting in timely clinical management of the disease which then prevents and/or minimizes onset of complications [41]. There is however a lack of data to fully investigate these trends in diabetes complications in high income countries and almost no data from other high-risk areas of the world [58].

The socio-economic implications of a high burden of diabetes and related complications for LMICs are immense, as the management of these conditions can consume vast amounts of household and governments’ spending [59]. The younger age at which type 2 diabetes is occurring in people in LMICs means that complications from diabetes threatens economic productivity and livelihoods of families and communities [54]. The inability to work has a knock-on effect on access to care. Most people in LMICs access care largely by out-of-pocket payments due to weak health systems that cannot meet health care demands. Therefore, failure to earn an income becomes a barrier to accessing healthcare, increasing morbidity and mortality.

From a clinical perspective, the high prevalence of diabetes complications at time of diagnosis suggests a need to improve early identification of people with undiagnosed diabetes. Several studies have evaluated targeted screening for type 2 diabetes and demonstrated effectiveness in identifying undiagnosed people who had a considerable prevalence of microvascular complications [60], however evidence on the cost effectiveness of screening strategies remains unclear [61, 62]. In the context of multimorbidity and increasing service integration, clinic based opportunistic screening particularly targeting high risk groups, may be a feasible approach to increasing early diagnosis of people with type 2 diabetes. However further evidence is needed, particularly on the cost effectiveness of these approaches, in resource limited settings.

### Strengths and limitations

According to our knowledge, this systematic review is among the first to estimate the prevalence of micro and macrovascular complications of diabetes in LMICs. The majority of studies included in this review enrolled large numbers of participants, improving generalizability of findings to populations from which participants were drawn and improving precision of proportions we reported. We only included articles published in peer reviewed journals, with >80% of the articles receiving a methodological quality rating of “fair/good”, potentially making our findings more robust. However, our review has some important limitations to consider.

The substantial interstudy heterogeneity observed in our study required a formal assessment of potential sources of variability such as gender, age, and type of health facility. However, efforts to explore the source of this variability statistically, were challenging due to paucity of studies in subgroup analyses. Potential reasons for the heterogeneity observed in these findings may relate to different screening procedures for these conditions, differences in definitions and methods of diagnosis in each study, differences in subjects’ ethnicities and their genetic predisposition to diabetes, especially considering that most of our eligible articles (73%) were from Asia, where the socio-economic profiles of countries within the region can differ widely. Even within the LMIC spectrum itself, geographic differences are anticipated with respect to quality of life, access to and quality of healthcare services. The prevalence of co-morbidities like hypertension and high cholesterol, and the extent to which these are diagnosed and treated may also have an impact on differences seen in prevalence of some microvascular and macrovascular complications [46]. This may be a contributing factor to some of the variability observed in this study.

Finally, the high interstudy variability observed may be due to differences in time frames in which the studies were carried, where the publication period for our eligible studies was from 1989–2020. We acknowledge that language restrictions in our inclusion criteria may have limited the scope of the search. Additionally, grey literature formed part of the exclusion criteria. Further work on this topic can include grey literature to increase the review’s comprehensiveness and timeliness to foster a balanced picture of the available evidence [63].

## Conclusion and recommendations

The prevalence of micro and macrovascular complications of diabetes at the time of diagnosis appears higher in LMICs compared to high income countries, however the high heterogeneity observed makes firm conclusions challenging. Our findings suggests that people in LMICs have a higher burden of undiagnosed diabetes complications. Further investigation of cost-effective ways for early identification and treatment of people with diabetes (or prediabetes) to reduce the associated morbidity and mortality is needed. Screening for complications at time of diagnosis should become routine practice to provide opportunity for timely intervention.

## Supporting information

S1 Checklist (DOCX)

S1 Text (DOCX)

S1 Table (XLSX)

S2 Table (XLSX)

S3 Table (XLSX)

S4 Table (XLSX)

S5 Table (XLSX)

S6 Table (XLSX)

S7 Table (XLSX)

S8 Table (XLSX)

S1 Data (XLSX)
