# Supplementary material for: Prevalence of microvascular and macrovascular complications of diabetes in newly diagnosed type 2 diabetes in low-and-middle-income countries: A systematic review and meta-analysis
Source: PLOS Glob Public Health. 2022 Jun 15;2(6):e0000599. doi: 10.1371/journal.pgph.0000599 (PMC10021817; doi:10.1371/journal.pgph.0000599)
Supplement: S1 Text — (DOCX) [file pgph.0000599.s002.docx]

**Information sources**

| **Medline was searched using the search strategy below from the date of creation of the database to 05/07/2020, for relevant abstracts of articles.** | | | |
| --- | --- | --- | --- |
| **Searches** | **Search combinations** | **Search terms** | **Number of hits** |
| S1 |  | MH Diabetes mellitus+ OR MH Diabetes mellitus, Type 2+ OR Diabetes mellitus, Type 1+ | **375,578** |
| S2 |  | Diabetes mellitus OR diabetes mellitus type 2 OR diabetes mellitus type 1 OR diabetes OR type 2 diabetes OR type 2 diabetes mellitus OR diabetic* OR type 2 diabetic* OR dysglycemia OR dysglycaemia OR hyperglycemia OR hyperglycaemia OR glucose OR insulin resistance OR insulin OR hyperinsulinemia OR hyperinsulinaemia | **1,106,256** |
| S3 | S1 OR S2 |  | **1,106,256** |
| S4 |  | new* diagnos* OR recent* diagnos* OR first* diagnos* OR newly-diagnosed OR time of diagnos* OR recently-diagnosed | **91,430** |
| S5 |  | MH diabetes complications+ | **193,369** |
| S6 |  | microvascular complication* OR macrovascular complication* OR microvascular OR macrovascular OR chronic complication* diabet* complication* OR diabetic retinopathy OR retinopathy OR maculopathy OR diabetic nephropathy OR nephropathy OR microalbuminuria OR macroalbuminuria OR albuminuria OR diabetic kidney disease OR diabetic neuropathy OR neuropathy OR sensory neuropathy OR peripheral neuropathy OR diabetic peripheral neuropathy OR peripheral arterial disease OR diabetic foot OR atherothrombo* OR myocardial infarction OR ischaemic heart disease OR stroke | **693,012** |
| S7 | S5 OR S6 |  | **744,294** |
| S8 | S3 AND S4 AND S7 |  | **2,334** |
| **CINAHL was searched using the search strategy below from the date of creation of the database to 05/03/2020, for relevant abstracts of articles.** | | |  |
|  |  |  |  |
| **Searches** | **Search combinations** | **Search terms** | **Number of hits** |
| S1 |  | MH Diabetes mellitus+ OR MH Diabetes mellitus, Type 2+ OR Diabetes mellitus, Type 1+ | **174, 403** |
| S2 |  | Diabetes mellitus OR diabetes mellitus type 2 OR diabetes mellitus type 1 OR diabetes OR type 2 diabetes OR type 2 diabetes mellitus OR diabetic* OR type 2 diabetic* OR dysglycemia OR dysglycaemia OR hyperglycemia OR hyperglycaemia OR glucose OR insulin resistance OR insulin OR hyperinsulinemia OR hyperinsulinaemia | **306,427** |
| S3 | S1 OR S2 |  | **307,163** |
| S4 |  | new* diagnos* OR recent* diagnos* OR first* diagnos* OR newly-diagnosed OR time of diagnos* OR recently-diagnosed | **28,518** |
| S5 |  | microvascular complication* OR macrovascular complication* OR microvascular OR macrovascular OR chronic complication* diabet* complication* OR diabetic retinopathy OR retinopathy OR maculopathy OR diabetic nephropathy OR nephropathy OR microalbuminuria OR macroalbuminuria OR albuminuria OR diabetic kidney disease OR diabetic neuropathy OR neuropathy OR sensory neuropathy OR peripheral neuropathy OR diabetic peripheral neuropathy OR peripheral arterial disease OR diabetic foot OR atherothrombo* OR myocardial infarction OR ischaemic heart disease OR stroke | **280,649** |
| S6 | S3 AND S4 AND S5 |  | **658** |
| **Global health was searched using the search strategy below from the date of creation of the database to 05/03/2020, for relevant abstracts of articles.** | | | |
| **Searches** | **Search combinations** | **Search terms** | **Number of hits** |
| S1 |  | Diabetes mellitus OR diabetes mellitus type 2 OR diabetes mellitus type 1 OR diabetes OR type 2 diabetes OR type 2 diabetes mellitus OR diabetic* OR type 2 diabetic* OR dysglycemia OR dysglycaemia OR hyperglycemia OR hyperglycaemia OR glucose OR insulin resistance OR insulin OR hyperinsulinemia OR hyperinsulinaemia | **255,632** |
| S2 |  | new* diagnos* OR recent* diagnos* OR first* diagnos* OR newly-diagnosed OR time of diagnos* OR recently-diagnosed | **16,100** |
| S3 |  | microvascular complication* OR macrovascular complication* OR microvascular OR macrovascular OR chronic complication* diabet* complication* OR diabetic retinopathy OR retinopathy OR maculopathy OR diabetic nephropathy OR nephropathy OR microalbuminuria OR macroalbuminuria OR albuminuria OR diabetic kidney disease OR diabetic neuropathy OR neuropathy OR sensory neuropathy OR peripheral neuropathy OR diabetic peripheral neuropathy OR peripheral arterial disease OR diabetic foot OR atherothrombo* OR myocardial infarction OR ischaemic heart disease OR stroke | **92,696** |
| S4 | S1 AND S2 AND S3 |  | **296** |
